# Methyl bromide fumigation and delayed mortality: safe trade of live pests?

**DOI:** 10.1007/s10340-014-0573-7

**Published:** 2014-03-07

**Authors:** C. B. Phillips, I. I. Iline, M. Novoselov, M. R. McNeill, N. K. Richards, C. van Koten, B. P. Stephenson

**Affiliations:** 1 AgResearch, Lincoln, Private Bag 4749, Canterbury, 8140 New Zealand; 2 Ministry for Primary Industries, PO Box 2526, Wellington, 6140 New Zealand

**Keywords:** Methyl bromide, Insect mortality, ATP, Phytosanitary inspection, Time to die

## Abstract

Live organisms intercepted from treated commodities during phytosanitary inspections usually arouse suspicions of treatment failure, sub-standard treatment application, or post-treatment infestation. The additional possibility that some treatments could kill slowly, meaning commodities might be inspected before pests have succumbed, is seldom considered for treatments other than irradiation. We used a novel biochemical viability assay to measure delays between methyl bromide fumigation and mortality of dipteran eggs, and evaluated the correspondence between egg viability and egg morphological features. Our experimental conditions simulated shipping of rock melons from Australia to New Zealand by sea and air. No eggs survived fumigation, but they took 3–20 days to die, whereas phytosanitary inspections of rock melons occur within 2–7 days. Delays were not influenced by methyl bromide concentration, but were significantly lengthened by cooler storage temperatures. Methyl bromide’s preservative effects delayed degradation of egg morphology, so the biochemical assay detected mortality long before morphological signs of egg death appeared. The results show that commodities subjected to effective methyl bromide treatments are at risk of being inspected before all pests have either died, or started to exhibit morphological signs of death. This could cause commodities to be unnecessarily rejected by quarantine authorities. Better methods than inspection for live pests are needed to assist authorities to gain assurance that treated commodities have been effectively disinfested. These could be developed by exploiting biochemical responses of pests and commodities to treatments.

## Introduction

Organisms discovered during phytosanitary inspections may be assessed for viability, both to evaluate quarantine risks and to monitor the efficacy of phytosanitary measures (Hallman et al. 2010). Detections of live pests can result in commodities being re-treated, re-shipped, or destroyed, which create economic losses as well as negative environmental effects due to commodity wastage and increased use of pesticides and transport. Usually, action is taken against an infested commodity even if it is treatment-certified (Hallman et al. 2010). An exception involves irradiated commodities because pests are known to survive for a period following irradiation, so authorities must rely on certification rather than inspection to verify treatment efficacy (Hallman et al. 2010; Cannon et al. 2012).

Detections of live organisms in commodities previously subjected to treatments except irradiation are vexing for exporters and quarantine authorities because organisms present during treatment are expected to have died (Hallman et al. 2010); they arouse suspicions of treatment failure, sub-standard treatment application, or post-treatment infestation. However, another seldom considered possibility is that pests are still succumbing to a slow acting treatment at the time of inspection. This occurred following fumigation of grapefruit with 40 g/m^3^ methyl bromide; no larvae of *Anastrepha ludens* (Loew) (Diptera: Tephritidae) pupariated, but some continued to move for several days afterward, whereas fruit inspections occurred 1 day afterward (Hallman and Thomas 2011).

There are other published indications that methyl bromide is slow acting. Cheetham (1990) studied *Cydia pomonella* (L.) (Lepidoptera: Tortricidae) eggs following methyl bromide fumigation at 8, 24 and 48 g/m^3^ for 2 h at 21 °C. The absence of mitotic activity indicated that most eggs treated with the highest dose had died after 20 h, and all were certainly dead after 96 h. However, many eggs treated with the two lower doses may still have been alive after 96 h, though they showed cellular abnormalities indicating mortal damage. Yokoyama et al. (1988) also monitored *C. pomonella* eggs after methyl bromide fumigation; a few 1-day-old embryos continued development for 15–16 days, but these never hatched (see also Boczek et al. 1975). Forney et al. (1991) measured declines of adenosine triphosphate (ATP) in eggs of *Asynonychus godmani* Crotch (Coleoptera: Curculionidae) after fumigation with 80 g/m^3^ methyl bromide for 2 h at 20 °C. ATP concentrations declined by 95 % after 2 h in eggs killed by freezing in liquid N for 30 s, but only by 50 % after 24 h in fumigated eggs.

If long delays between methyl bromide fumigation and pest mortality are common, then effectively treated commodities could be unnecessarily rejected by quarantine authorities due to the presence of live, but lethally treated and dying pests. Avoiding unnecessary rejections would require development of alternatives to inspection to provide quarantine authorities with confidence that commodities are safe and any live pests present have been lethally treated. Methods other than certification would also be helpful for assuring phytosanitary safety of irradiated commodities (Hallman et al. 2010; Cannon et al. 2012).

Delays between treatment and pest mortality have seldom been measured because the precise time when mortality occurs is difficult to determine, especially when organisms are immobile even when alive (e.g., eggs, pupae, and scale insects). However, it recently became practical to make these measurements with the advent of a biochemical assay for assessing arthropod viability, which exploits rapid postmortem degradation of ATP for differentiating between living and dead specimens (Phillips et al. 2013). It is based on techniques for staining hexokinase (EC 2.7.1.1) (Murphy et al. 1996) and for detecting ATP (Lamprecht and Trautschold 1974). It signals the presence of ATP in a live specimen by producing a strongly colored formazan, but produces no color change when ATP is either greatly depleted or absent, and the organism is dead. It has been comprehensively validated during more than 15,000 assays of over 80 arthropod species (Phillips et al. 2013).

Delays between methyl bromide fumigation and mortality should be influenced by factors similar to those that influence fumigation efficacy (Bond 1984): pest species (Armstrong and Whitehand 2005), life stage (Hallman and Thomas 2011), commodity (Yokoyama et al. 1990), fumigation rate (Armstrong and Whitehand 2005), fumigation temperature (Armstrong and Whitehand 2005), and post-fumigation storage temperature. Fumigation efficacy is increased by conditions that promote insect respiration such as warm temperatures, slightly elevated CO_2_ and reduced O_2_ (Bond 1984; Cotton 1932). We could not find any studies that investigated the influence of post-fumigation storage temperature on delay to mortality (c.f. mortality rate), but expected it would decrease with increasing post-fumigation storage temperature.

Here, we used a biochemical assay (Phillips et al. 2013) and egg hatch observations to measure delays between methyl bromide fumigation and mortality of insect eggs under conditions that simulated importation of rock melons from Australia to New Zealand by sea and air. The experiments were conducted at the request of New Zealand’s quarantine authority, Ministry for Primary Industries (MPI), because it regularly intercepts and assesses the viability of insect eggs from imported produce that is certified as having been fumigated with methyl bromide (e.g., Keyes 2010). These eggs often appear to be alive, and MPI suspected that delays between methyl bromide fumigation and egg mortality were the explanation. Interceptions of other stationary life stages (e.g., pupae) and sessile arthropods (e.g., scale insects and whiteflies) present similar risk assessment difficulties. MPI’s quarantine standards for imported fresh produce are defined in Ministry of Agriculture and Forestry (2013), and procedures for diagnosing intercepted specimens are given in Ministry of Agriculture and Forestry (2000).

We studied the egg stage because its viability is difficult for quarantine authorities to assess using criteria based on morphology and movement, and it is found during phytosanitary inspections (Armstrong and Ball 2005; Ebina et al. 2004; Forney et al. 1991; Keyes 2010). We used a dipteran because species from this order are often intercepted throughout the world (McCullough et al. 2006; Roques and Auger-Rozenberg 2006) and were also problematic for MPI. Diptera families prominent in published interception records include Agromyzidae and Tephritidae (Roques and Auger-Rozenberg 2006; Haack 2001), Cecidomyiidae (Haack 2001; Iwaizumi et al. 2007), Culicidae (Derraik 2004; Medlock et al. 2012), and Lonchaeidae (Haack 2001). Diptera families frequently intercepted by MPI that require viability assessment include Agromyzidae, Drosophilidae, Lonchaeidae, and Muscidae, which are found in association with fresh produce such as rock melons, capsicums, citrus fruits, and strawberries imported from Australia. Tephritidae are also intercepted by MPI, usually as eggs or early instar larvae (Armstrong and Ball 2005; Keyes 2010), but do not require viability assessments because they always invoke a regulatory response whether dead or alive (Ministry of Agriculture and Forestry 2000). Conducting experiments on exotic pests not present in New Zealand was impractical, so we used eggs of the house fly, *Musca domestica* L. (Diptera: Muscidae), because it was easily cultured to provide eggs of known age and quality for experiments.

We used a biochemical assay (Phillips et al. 2013) and egg hatch observations to measure delays between methyl bromide fumigation and mortality of insect eggs under conditions that simulated importation of rock melons from Australia to New Zealand by sea and air. MPI generally uses an unpublished protocol based on morphological characters for assessing egg viability (Richmond and O’Donnell 2005, unpublished). We compared morphological indicators of egg viability with biochemical assay results by conducting a morphological assessment of each egg before biochemically assaying it. Our results confirmed that methyl bromide is slow acting, and showed this is exacerbated by its preservative effect, which greatly defers the onset of morphological signs of death. Thus, delays to mortality were shorter when mortality was assessed biochemically rather than morphologically. However, even when measured biochemically, delays to mortality exceeded the intervals between treatment and inspection for rock melons imported from Australia.

## Materials and methods

Two experiments were conducted. Experiment 1 made preliminary evaluations of the effects of one methyl bromide fumigation rate and two post-fumigation storage temperatures to refine the methods to be used for a more comprehensive second experiment. Experiment 2 measured delays in mortality following two fumigation treatments and four post-fumigation storage treatments.

Table 1 lists the treatments for Experiments 1 and 2, and provides the abbreviation hereafter used for each treatment. In Experiment 2, the 45g.7C-2d treatment simulated air freight of rock melons between Australia and New Zealand, and the 45g.7C-5d treatment simulated sea freight.

**Table 1:** Treatments, treatment abbreviations, number of eggs assessed for viability, and days to ≤0.05 survival from Kaplan–Meier analysis

Experiment	Fumigation rate (g/m^3^)	Post-fumigation storage (°C)	Treatment abbreviation	Eggs assessed for viability^a^	Days to ≤0.05 survival
1	nil	11	nil.11C	200	na^b^
	nil	21	nil.21C	200	na^b^
	40	11	40 g.11C	90	21
	40	21	40 g.21C	56	14
2	nil	10	nil.10C	309	na^b^
	nil	20	nil.20C	271	na^b^
	nil	7 °C for 2 days, then 20 °C	nil.7C-2d	501	na^b^
	nil	7 °C for 5 days, then 20 °C	nil.7C-5d	482	na^b^
	25	10	25g.10C	515	19.9
	25	20	25g.20C	234	7.8
	45	10	45g.10C	482	18.8
	45	20	45g.20C	326	8.9
	45	7 °C for 2 days, then 20 °C	45g.7C-2d	282	8.9
	45	7 °C for 5 days, then 20 °C	45g.7C-5d	389	14.7

^a^Totals of the counts shown in Fig. 1

^b^Survival probability did not decline to 0.05

### Viability assessment methods

In both experiments, data on egg viability were obtained using three methods: observing hatching, making morphological assessments, and conducting biochemical viability tests. Each hatched egg was scored as alive. Each unhatched egg was morphologically assessed, then biochemically assayed and classified as alive or dead based on the biochemical result. Details of these methods are given below.

#### Hatching

Eggs were examined every day; the number hatched was recorded and larvae were removed from Petri dishes.

#### Morphological

We used a modification of a morphological assessment protocol that was developed by MPI to assist its diagnosticians to determine the life state of sessile or otherwise immobile life stages of invertebrates (Richmond and O’Donnell 2005, unpublished). It gives separate criteria for eggs, pupae, and Coccoidea. For eggs, seven criteria are listed: looking for embryo movement in slide mounted eggs with a compound microscope; evaluating egg decomposition/fungal growth, egg color, egg turgidity, embryo development, and embryo condition using a stereo microscope; and looking for larvae near eggs. Embryo movement confirms eggs are living, and decomposition confirms they are dead. When no embryo movement is observed, but other characteristics of eggs appear normal, then eggs are considered live (Richmond and O’Donnell 2005, unpublished). Before the experiment, MPI provided us training to ensure we applied its protocol proficiently. We evaluated all of MPI’s criteria except for embryo movement because time limitations while processing eggs precluded us from preparing slide mounts.

#### Biochemical

We followed the biochemical viability assay method of Phillips et al. (2013). Clean fine-tipped forceps were used to macerate individual *M. domestica* eggs at room temperature in 6 μl of stain (final concentrations of 4 U/ml hexokinase, 0.1 M Tris pH 7.8, 0.03 mM mPMS, 0.6 mM MTT, 0.2 % Triton X-100, 0.4 mM NAD, 2 U/ml G6PGD, 2 U/ml 6PGD, 1 mM MgCl_2_, and 5 mM d-glucose) in 0.04 ml microtubes that had been cut in rows from 384-well plates typically used for amplifying DNA (PCR plates; Thermo Scientific, Switzerland, AB-1384). Live eggs contain sufficient ATP for tetrazolium salt (MTT) to be reduced to a purple formazan dye within 10 min of starting an assay, but dead eggs do not (Phillips et al. 2013). Although visual interpretations of assay results are reliable, to ensure objectivity and facilitate data analysis, we quantified each color reaction using a standardized procedure for digitally photographing each tube after 10 min of incubation at room temperature (Phillips et al. 2013). We used Image analysis software (ImageJ v. 1.44, United States National Institute of Health, http://rsb.info.nih.gov/ij/) to measure the mean pixel value of the blue channel divided by the mean pixel value of the green channel (B:G) in the image of each staining reaction (Phillips et al. 2013). B:G declines with ATP concentration and is closely correlated with visual color observations (Phillips et al. 2013). Eggs producing test reactions with B:G ≥0.97 were classified as alive, and those with B:G <0.97 were classified as dead; this threshold was determined by extensive testing described in Phillips et al. (2013).

### Experiment 1

#### Insects

We purchased *M. domestica* eggs from a commercial producer (Biosuppliers New Zealand, www.biosuppliers.com), who delivered them in an insulated box containing a frozen cooler pad. To maintain the high humidity needed for egg survival, they were transferred to Petri dishes (90 mm diameter by 15 mm deep) lined with moistened filter papers. Eggs were kept for a maximum of 12 h at 10 °C before treatment.

#### Fumigations

Eggs were transported to and from the fumigation facility in insulated boxes, and data loggers (Gemini Tinytag Ultra 2) recorded temperatures within the boxes every 10 min during transport. To ensure treated and control populations were subjected to identical conditions, control eggs were transported with the treated eggs to and from the fumigation facility, but without exposing them to fumigant.

A commercial fumigation company located in Christchurch, approximately 20 min by car from our laboratory, was contracted to treat the eggs. Treated eggs were fumigated in a single chamber, temperature was recorded every 10 min, but fumigant concentration was not monitored.

#### Post-fumigation storage

Upon return to our laboratory, eggs were kept in controlled temperature chambers, using one chamber per temperature (7, 10 and 20 °C; Table 1), and data loggers were used to record temperatures in each chamber every 10 min during storage. Within chambers, the positions of Petri dishes containing eggs were rotated at approximately 24 h intervals to reduce the effect of any temperature stratification. Filter papers within the Petri dishes were regularly checked, and 0.1 ml of sterile water was applied whenever they began to dry.

#### Subsampling of eggs for viability assessment

The first subsamples of eggs for making viability assessments were taken approximately 3 h after fumigation, then every 24–48 h. Subsamples for viability testing each consisted of ≥7 eggs, while additional eggs (*n* ≥ 100) were maintained to record hatching.

### Experiment 2

#### Insects

We cultured *M. domestica* using established methods (Sawicki 1964; Shipp and Osborn 1967). Oviposition and larval development occurred on paper tissues soaked in a solution containing bran and milk powder. Oviposition was scheduled by withholding protein from the flies’ diet and preventing access to an oviposition substrate until eggs were needed. No evidence of disease or parasitism was observed.

Egg viability was checked 14 and 4 days before the start of the experiment by counting the proportion of ≥200 subsampled eggs that hatched.

 Petri dishes (60 mm diameter by 14 mm deep) were prepared c. 12 h before the experiment began by labeling them with a treatment name, lining them with three circular filter papers (Whatman grade 4, 50 mm), then pipetting 1 ml of sterile water onto the paper. Over 250 Petri dishes were prepared to make provision for all subsequent subsampling. To reduce contamination by microorganisms, dishes were prepared in a laminar flow cabinet, and sterile water was used to moisten the filter papers. Dishes were stored at 10 °C until required. Eggs <6 h old were collected from the culture, rinsed in 10 °C sterile water, and then stored at 10 °C to impede their development. Batches of the <6 h old eggs were then retrieved from the 10 °C chamber, and 30 randomly selected eggs were transferred to each dish using a size 0 fine art paint brush. Petri dishes, each containing 30 eggs, were returned to 10 °C pending transport to the fumigation facility. The maximum time any egg was stored at 10 °C prior to transport was 12 h, and the maximum time eggs were kept at room temperature, while transferring them to Petri dishes was approximately 1 h. To help maintain high humidity, Petri dishes were stored in larger sealed containers lined with three sheets of Whatman grade 1 chromatography paper. Approximately, 15 ml of sterile water was used to initially saturate the container liners, and these were then kept moist throughout the experiment by applying 1–5 ml of sterile water as required.

#### Fumigations

*Musca domestica* eggs were transported in insulated boxes, which were loaded with bottles of water preconditioned to the required temperatures to moderate temperature fluctuations. Data loggers (Gemini Tinytag Ultra 2) recorded temperatures within the boxes every 10 min during transport. To ensure treated and control populations were subjected to identical conditions, control eggs were transported with the treated eggs to and from the fumigation facility, but without exposing them to fumigant.

Fumigations were performed at a fumigation research facility located in Palmerston North, which is approximately a 40 min drive plus a 1 h flight from our laboratory. Each fumigation treatment was split across three separate fumigation chambers. Chambers and eggs were maintained at 20 °C for approximately 20 min immediately before starting the fumigations. Fumigant concentration was measured using a pre-calibrated “MBContainIR Multizone” eight-port analyser (Spectros Instruments Inc., Hopedale, MA 01747, USA), which has a sensitivity of 0.16 g/m^3^ and accuracy of ±4 %. Measurements were obtained from each of the six chambers at approximately 12 min intervals.

Temperatures were recorded at approximately 9 min intervals in one of the three chambers in which 25 g/m^3^ of fumigant was applied, and in two of the three 45-g/m^3^ chambers. We verified that chambers without temperature loggers provided results similar to those with temperature loggers using methods described in the “Survival analysis” section below. Lids were removed from Petri dishes containing eggs, in both the treated and control groups, for the duration of the fumigation. Control eggs were kept at 20 °C while treated eggs were being fumigated.

#### Post-fumigation storage

Eggs were stored as previously described for Experiment 1.

#### Subsampling of eggs for viability assessment

Eggs were subsampled every 24 h, except on days 4–8 when they were subsampled twice every 24 h. Sampling frequency was increased on days 4–8 to maximize data resolution during the period when mortality rates were expected to change most quickly based on results from the most effective treatment in Experiment 1. Subsampling involved removing one Petri dish containing 30 eggs from each treatment, counting the number of eggs that had hatched, recording the morphological condition of up to 15 unhatched eggs, and then subjecting those same unhatched eggs to biochemical assays. Subsampled dishes and insects were then removed from the experiment.

On each sampling occasion, if no survivors were found in the initial subsample of 15 eggs, then all of the eggs remaining in the same dish were also assayed. If none were alive, then testing was continued using further dishes from the same treatment until mortality had been confirmed in 100 % of at least 50 eggs. If a low rate of survival was detected (e.g., one survivor from 40 eggs), then further assaying of that treatment was postponed until the following day when the process was repeated. Daily subsampling from each treatment continued either until no survivors were detected during testing of at least 50 eggs, or until all eggs had hatched.

Once mortality of 100 % of eggs in a treatment had been confirmed, biochemical assaying ceased, but any eggs remaining from that treatment were maintained at their designated storage temperature so morphological assessments could continue. These morphological assessments generally occurred once each day until further assessment became uninformative due to eventual decomposition of the eggs. Unlike the earlier morphological assessments and biochemical assays, when subsampled Petri dishes were removed from the experiment, the postmortem morphological assessments involved repeated observations of the same eggs.

### Temperature specifications

The 45g.7C-2d and 45g.7C-5d treatments in Experiment 2 (see Table 1 for definitions of treatment abbreviations) aimed to simulate the temperatures used for air and sea freight of rock melons from Australia to New Zealand. To evaluate how closely each experiment met its temperature specifications, we modified the standard calculation of degree days (°d) for insect development (Nietschke et al. 2007) by summing each experiment’s absolute deviations from its specified temperatures, rather than the actual deviations from an insect’s development threshold. The experimental deviation from the temperature specification was calculated as *days* × (*actual temperature* − *specified temperature*). For example, in Experiment 1, the actual fumigation was at 19.4 °C for 2 h, while 20 °C for 2 h was specified. 2 h is 0.083 days, so the deviation is 0.083 days × (19.4–20 °C) equals −0.05 days, and the absolute deviation is 0.05 days. To obtain the total deviation for each experiment, the absolute values of the deviations from across all treatments, and phases of the experiment (i.e., fumigation, transport back to laboratory, and post-fumigation storage) were summed up to the time at least 95 % of fumigated eggs had died. This total was expressed as a percentage of the degree days that would have accumulated if all temperature specifications had been perfectly met.

### Survival analysis

Two statistical approaches were used to estimate survival times. The first was the Kaplan–Meier method which is a standard non-parametric statistical method for survival time analysis (Crawley 2007). The second involved parametric survival analysis where different parametric curves (Exponential, Gaussian, Poisson, Log Normal, Logistic, and Weibull) were fitted to the data, and the best fitted curve was determined using either the Anderson–Darling (AD) statistic or the Akaike information criterion (AIC). To analyze Experiment 2, a generalized linear model (GLM) with Poisson errors was also fitted, and its residual deviance and AIC were compared to the other parametric models. The results present Kaplan–Meier analyzes for both experiments and adds parametric analyzes only where they provide pertinent additional information.

In Experiment 2, to verify that the three fumigation chambers in which temperatures were not monitored provided equivalent results to those from which temperature data were recorded, survival of eggs from different chambers that had been subjected to the same treatment were compared. Thus, the results from the three 25-g/m^3^ chambers were compared within each of the 25g.20C and 25g.10C treatments, while those from the three 45-g/m^3^ chambers were compared within each of the 45g.20C, 45g.7C-2d, 45g.7C-5d, and 45g.10C treatments. The parametric model showing the best fit was then chosen for further analysis. Where this was a GLM, the significance of fumigation chamber was tested using *χ* ^2^ statistic by comparing models with and without fumigation chamber as a factor (Crawley 2007).

Analysis was conducted using R (R Core Team 2012) and the R package “survival” (Therneau 2012). Figures were prepared using the R package “ggplot2” (Wickham 2009).

### Comparison between assays and morphological evaluations

To measure delays between treatment and egg mortality, only results from biochemical assays and hatching observations were used. However, to evaluate the performance of morphological criteria for assessing viability, a second Kaplan–Meier survival analysis was conducted where eggs exhibiting at least one morphological sign of death (decomposition, fungal growth, discoloration, or reduced turgidity) were classed as dead. The absence of a visible larva within an egg was not a reliable sign of egg death (see “Visibility of larvae within eggs” section in the “Results”) and was not used. The survival analysis based on morphological criteria included observations of individual eggs made just before they were assayed, plus repeated observations of the same eggs made after 100 % mortality of a treatment had been confirmed.

## Results

### Alignment with temperature and fumigant specifications

In Experiment 1, temperatures were close to specification; the largest deviation was for 20 min during transport from the fumigation facility back to our laboratory when eggs specified for storage at 20 °C traveled at a mean temperature (±SD) of 17.3 ± 0.3 °C. However, the short period of this deviation (0.3 h) compared to the overall interval from the start of fumigation to eventual ≥95 % mortality of fumigated eggs, and the good precision of the controlled temperature chambers used to store the insects post-fumigation (mean ± SD of 10.7 ± 0.2 °C for the 10 °C chamber, and 20.8 ± 0.7 °C for the 20 °C chamber), meant there was only an overall 5.3 % deviation from the specified temperatures.

In Experiment 2, temperatures were close to those specified; the largest deviation was for 4 h during transport from the fumigation facility back to our laboratory when eggs specified for storage at 10 °C traveled at a mean temperature of 13 ± 1.4 °C. The overall deviation from the specified temperatures was 1.1 %.

In Experiment 2, methyl bromide concentration was measured ten times during the 2-h fumigation in each of the six fumigation chambers. The overall mean (±SD) for the 25-g/m^3^ treatment was 24.6 ± 2.7 g/m^3^, and for the 45-g/m^3^ treatment was 45.6 ± 17.1 g/m^3^.

### Experiment 1 survival times

**Fig. 1:**
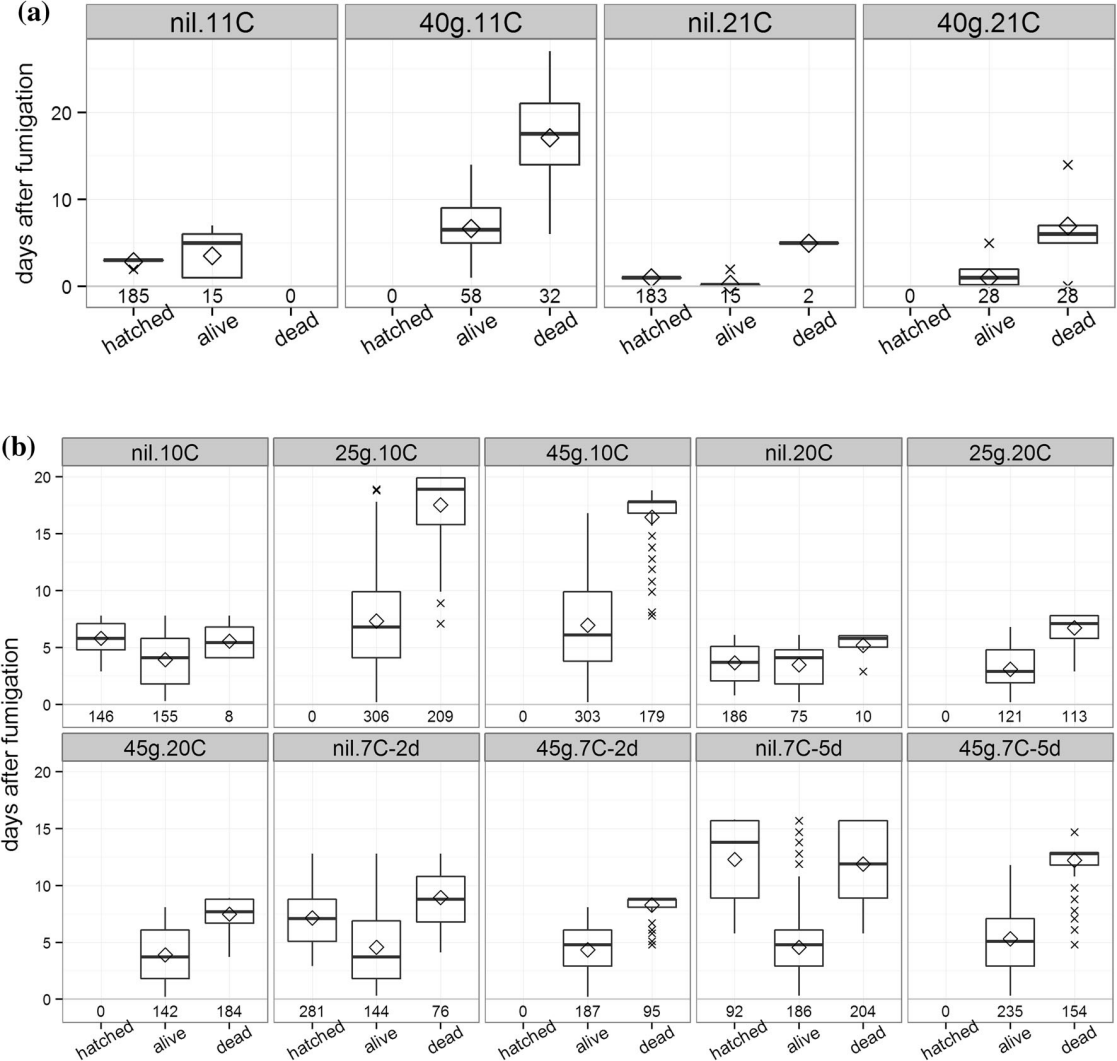
Box plots of **a** Experiment 1 and **b** Experiment 2 showing the number of days after fumigation when *M. domestica* eggs either “hatched”, or were “assayed alive” or “assayed dead”, by treatment. *Lower* and *upper horizontal lines* of *each box* show first and third quartiles, respectively. *Whiskers* extend to the highest and lowest values that are within 1.5 times the distance between the first and third quartiles. *Diamonds* show means, “*x*” show outliers, and *numbers* are counts of observations

Overall, 546 eggs either hatched or were biochemically assayed (Table 1). No fumigated eggs hatched, but many remained alive for varying periods following treatment. Figure 1a shows, for each treatment, box plots of eggs hatched, eggs assayed as alive, and eggs assayed as dead versus days post-fumigation. It also gives counts of observations.

In the controls (nil.11C and nil.21C), 99.5 % of eggs either hatched or gave alive assay results, and hatching and alive assays persisted longer with 11 °C storage than 21 °C storage (Fig. 1a). In the treatments (40 g.11C and 40 g.21C), no eggs hatched, but they gave alive assays for means (±SE) of 6.7 ± 0.51 days with 11 °C storage and 1.0 ± 0.21 days with 21 °C storage (Fig. 1a). Fumigated eggs died after a mean of 17.1 ± 0.84 days with 11 °C storage, and after 6.97 ± 0.61 days with 21 °C storage (Fig. 1a).

Kaplan–Meier estimates of time required for survival of Experiment 1 eggs to decline below 0.05 are given in Table 1. Estimates for controls are not shown because all eggs stored at 10 °C hatched, so the survival probability was 1.0, and eggs stored at 20 °C also had very high survival probabilities with a Kaplan–Meier 95 % confidence interval (95 % CI) of 0.974–1.0. Control eggs survived significantly longer than fumigated eggs stored at the same temperature (logrank test, *p* < 0.001). The mean survival probability of fumigated eggs stored at 20 °C declined to 0.15 (95 % CI of 0.01–0.36) after 7 days, and to 0 after 14 days. The mean survival probability of fumigated eggs stored at 10 °C declined to 0.04 (95 % CI of 0.01–0.25) after 21 days, and to 0 after 27 days. The survival curves for these two treatments were significantly different (logrank test, *p* < 0.001).

### Experiment 2 Survival Times

In the test conducted 14 days before the start of Experiment 2, 85 % of 240 eggs hatched, and in the other conducted 4 days beforehand 83.5 % of 200 eggs hatched.

Days to <0.05 survival of eggs fumigated in chambers without temperature data were very similar to those of eggs fumigated in chambers with temperature data (data not shown), and no systematic bias in days to <0.05 survival was apparent between chambers. For each parametric analysis of within-treatment variation between chambers, a GLM with Poisson errors gave the best fit. The parametric analysis showed no significant differences between chambers in any treatment except 45g.10C, where survival of eggs from chamber six exhibited a slightly different relationships with days after fumigation to eggs from chamber five (*p* = 0.004). These were the two 45-g/m^3^ chambers for which temperature data were recorded.

Table 1 gives the numbers of eggs that either hatched or were biochemically assayed, for each treatment. No fumigated eggs hatched, but many remained alive for varying periods following treatment. Figure 1b shows, for each treatment, box plots of eggs hatched, eggs assayed as alive, and eggs assayed as dead versus days post-fumigation. It also gives counts of observations.

In the controls (nil.10C, nil.20C, nil.7C-2d, nil.7C-5d), egg mortality was <4 % when stored at 10 or 20 °C (18 dead from 580 eggs), but mortality increased to 15 % when stored at 7 °C for 2 days (76 dead from 501 eggs), and to 42 % when stored at 7 °C for 5 days (204 dead from 482 eggs; Fig. 1b). Mean delays to egg hatch and egg mortality both increased as storage temperature decreased (Fig. 1b). In the treatments, no fumigated eggs hatched, but they remained alive for means (±SE) that ranged from 3.4 ± 0.20 days in the 25g.20C treatment to 7.4 ± 0.28 days in the 25g.10C treatment (Fig. 1b). Mean delays between fumigation and death ranged from 6.7 ± 0.13 days in the 25g.20C treatment to 17.5 ± 0.20 days in the 25g.10C treatment (Fig. 1b). Figure 1b shows a much stronger effect of storage temperature than fumigation rate on delays between fumigation and mortality.

**Fig. 2:**
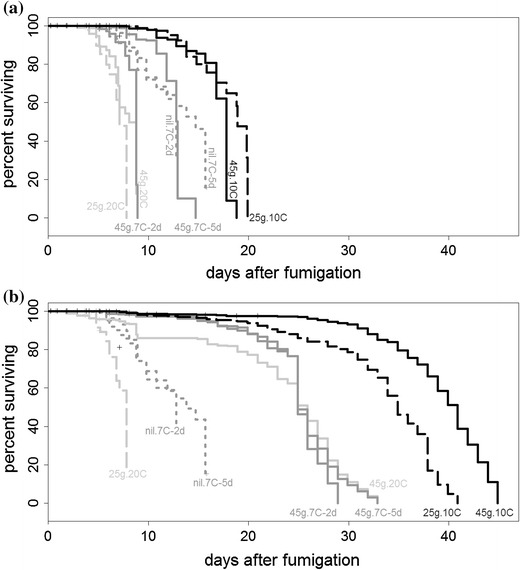
Experiment 2 Kaplan–Meier survival curves using observations of egg hatching plus **a** biochemical assay results, or **b** egg morphological attributes. *Dotted lines* show controls, *dashed lines* show 25-g/m^3^ fumigations, and *solid lines* show 45-g/m^3^. *Light gray lines* show post-fumigation storage at 20 °C, *medium gray* shows 7 °C for both 2 and 5 days, and *black* shows storage at 10 °C. Nil.10C and nil.20C omitted because survival probabilities remained high

The Kaplan–Meier survival curves calculated using biochemical assay results for eggs from each treatment in Experiment 2 are shown in Fig. 2a, and survival times are summarized in Table 1.

Control eggs stored at 10 and 20 °C had high mean survival probabilities (95 % CI) of 0.91 (0.84–0.99) and 0.81 (0.71–0.94), respectively, so their survival curves were omitted from Fig. 2a. Control eggs stored at 7 °C for 2 days also had a high mean survival probability of 0.30 (0.20–0.45), while those stored at 7 °C for 5 days had a mean survival probability of 0.15 (0.11–0.21).

For fumigated eggs, the period for mean survival probabilities to decline below 0.05 ranged from 7.8 days for 25g.20C eggs to 19.9 days for 25g.10C eggs (Fig. 2a; Table 1). The logrank test indicated that there were significant differences between some of the survival curves (*p* < 0.001, *χ* ^2^ = 2,309, 9 df), and these are examined more closely in the following parametric analysis.

A GLM with Poisson errors gave the best fit to the data (residual deviance = 1,525.7, 3,781 df, AIC = 4,181.7, log likelihood = –2,080) compared with the other models tested. It indicated that all but four treatments were significantly different from one another (*p* ≤ 0.05; Table 2). Two of the non-significant comparisons were 25g.10C c.f. 45g.10C (*p* = 0.85) and 25g.20C c.f. 45g.20C (*p* = 0.98), which confirmed the qualitative indication from Fig. 1b that there was no effect of fumigation rate on egg survival.

**Table 2:** Probabilities that treatments are the same from a GLM

	nil.20C	nil.10C	25g.20C	45g.20C	45g.7C-2d	nil.7C-2d	nil.7C-5d	45g.7C-5d	45g.10C
nil.10C	**0.073**								
25g.20C	<0.001	<0.001							
45g.20C	<0.001	<0.001	**0.983**						
45g.7C-2d	<0.001	<0.001	0.002	0.0006					
nil.7C-2d	0.027	<0.001	<0.001	<0.001	<0.001				
nil.7C-5d	<0.001	<0.001	<0.001	<0.001	0.001	<0.001			
45g.7C-5d	<0.001	<0.001	<0.001	<0.001	0.012	<0.001	**0.490**		
45g.10C	<0.001	<0.001	<0.001	<0.001	<0.001	<0.001	0.049	0.012	
25g.10C	<0.001	<0.001	<0.001	<0.001	<0.001	<0.001	0.026	0.006	**0.853**

Non-significant comparisons are shown in bold

A third non-significant comparison (Table 2) was between the nil.7C-5d and 45g.7C-5d treatments (*p* = 0.49). This confirmed that storage at 7 °C negatively influenced survival of control eggs to the extent that they died at similar rates to fumigated eggs stored at the same temperature. Nevertheless, 92 of the nil.7C-5d eggs and 281 of the nil.7C-2d eggs hatched, but no fumigated eggs did (Fig. 1b).

The final non-significant comparison was between the nil.20C and nil.10C treatments (*p* = 0.07) which reflects the high hatching rates and correspondingly low mortality rates recorded in both treatments (Fig. 1b).

**Fig. 3:**
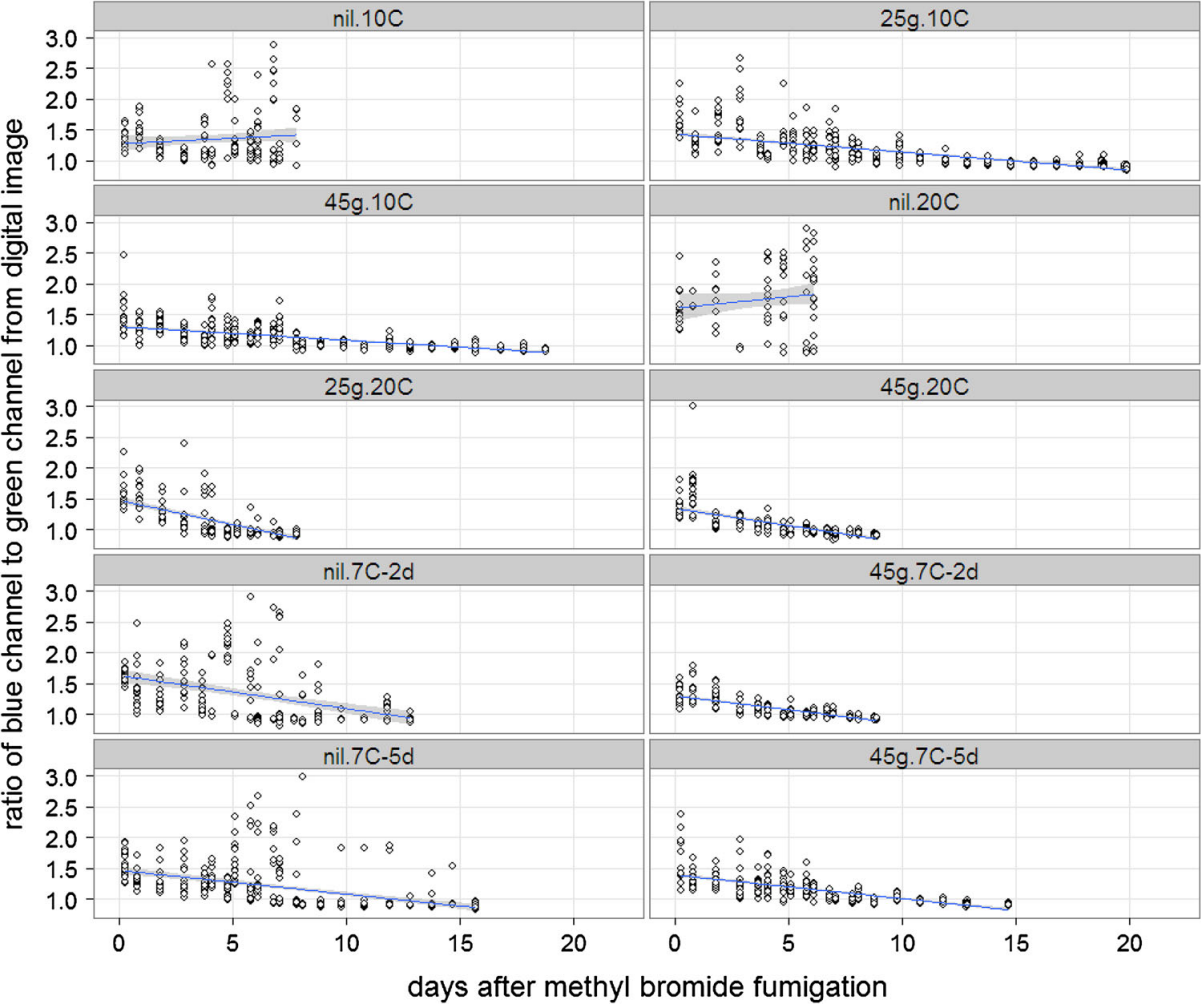
B:G ratios from individual *M. domestica* eggs subjected to different treatments in Experiment 2. *Lines* are linear regressions and *gray shading* is ±1 SE

Figure 3 shows B:G ratio, which is correlated with ATP concentration, versus time since fumigation for individual *M. domestica* eggs in Experiment 2. ATP concentrations in control eggs stored at 10 or 20 °C (“nil.10C” and “nil.20C” treatments), which mostly survived, increased with time. In contrast, ATP concentrations of control eggs stored at 7 °C (“nil.7C-2d” and “nil.7C-5d” treatments), which mostly died, declined with time. Similarly, ATP concentrations in fumigated eggs, which all died, also declined with time. As expected from results of biochemical viability assessments, rates of change of ATP concentrations were positively correlated with storage temperature (Fig. 3).

### Morphological variables

In Experiment 2, each egg’s morphological attributes were recorded immediately before assaying it. In these cases, it was possible to directly link an egg’s morphological data with its biochemical assay result, and such eggs were classified either as “assayed alive” or “assayed dead”. Subsampling of eggs for assessment both by morphology and by assay continued until mortality had been observed in 100 % of at least 50 eggs. After this time, biochemical assaying ceased, while the morphology of any remaining untested eggs was repeatedly assessed until the eggs decomposed. These eggs were known to be dead because they came from the same treatments in which 100 % mortality had already been observed; but, because they were not assayed, they were classified as “inferred dead” rather than “assayed dead”.

#### Egg color

Most eggs were scored as white, yellow, gray, or brown. Other colors such as pink or red were occasionally observed and were also noted. Healthy eggs of *M. domestica* are white so, to simplify description, all egg colors other than white were classified as “abnormal.” Eggs that exhibited obvious signs of fungal infection such as mycelia were also classified as “abnormal”.

**Fig. 4:**
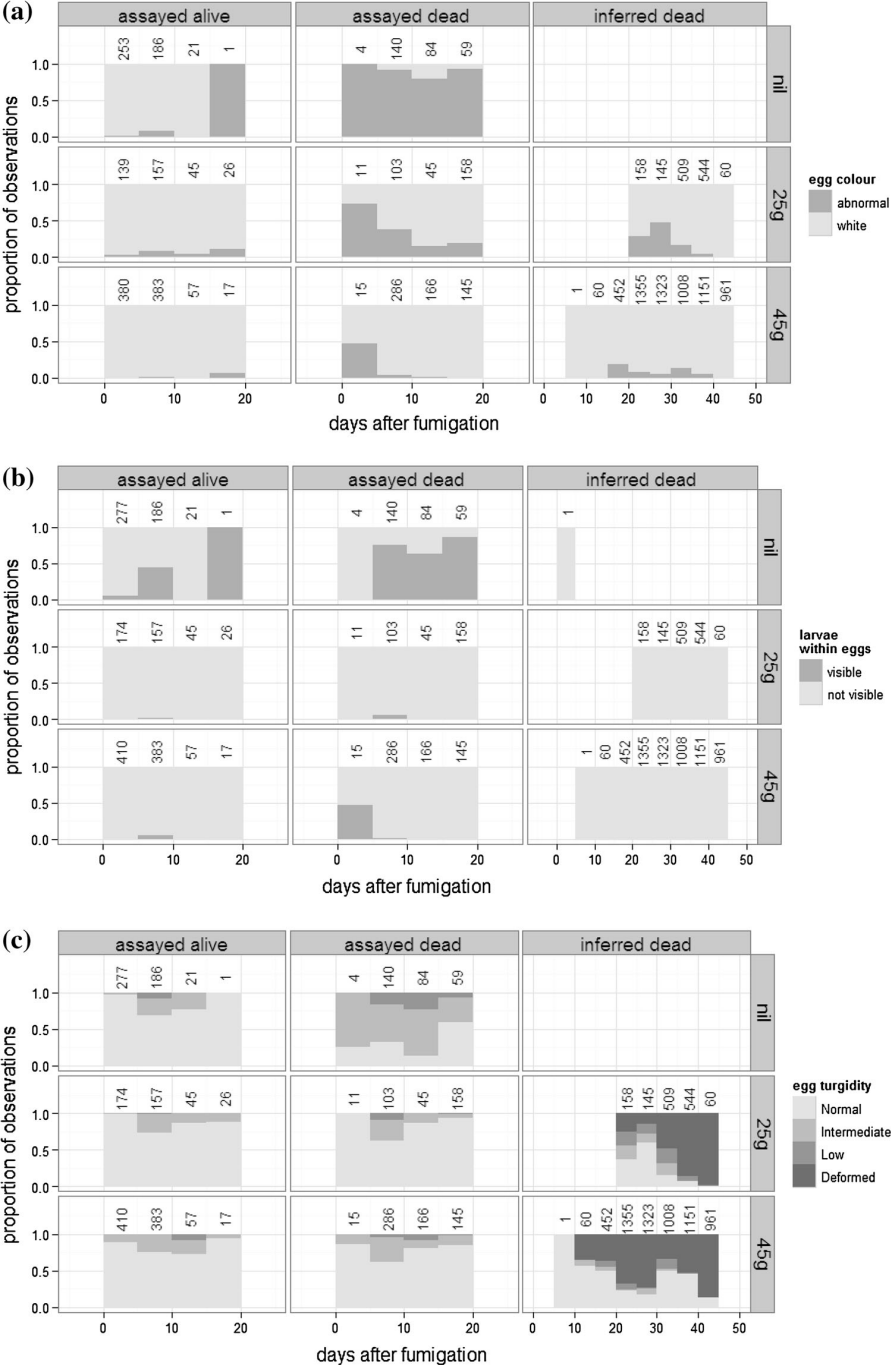
Experiment 2 frequencies of different *M. domestica* egg morphological attributes, versus days after fumigation, for “assayed alive,” “assayed dead,” and “inferred dead” eggs exposed to different concentrations of fumigant: **a** egg color, **b** the presence of visible larvae within eggs, and **c** egg turgidity. Observations have been grouped into intervals of 5 days, and the count of observations within each 5-day interval is shown above each column

Figure 4a shows the Experiment 2 frequencies of white and abnormal eggs, versus time after fumigation, for living and dead eggs exposed to different concentrations of fumigant. To simplify Fig. 4, data were binned into post-fumigation time intervals of 5 days. In the controls (“nil”, Fig. 4a), live eggs were mainly white and dead eggs abnormal. Fumigated eggs (“25 g” and “45 g”, Fig. 4a), however, were predominantly white, irrespective of whether they were assayed alive or assayed dead. Assayed dead eggs fumigated with 25 g/m^3^ methyl bromide had a higher proportion of abnormal eggs (26 % of 317 eggs) than assayed dead eggs fumigated with 45 g/m^3^ methyl bromide (3 % of 612 eggs; *χ* ^2^ = 116, df = 1, *p* < 0.001; Fig. 4a). Inferred dead eggs retained their normal coloration for up to 45 days after fumigation (Fig. 4a). Those retaining normal coloration for longest were from the 45g.10C treatment, which reflected the strong preservative effect of low storage temperature and high fumigation rate on normal egg color.

#### Visibility of larvae within eggs

Figure 4b shows the Experiment 2 frequencies of eggs that contained visible larvae, versus time after fumigation, for living and dead eggs exposed to different concentrations of fumigant. The presence of visible larvae exhibited a pattern similar to egg color. In the controls, larvae were visible only in 19 % of 485 assayed alive eggs, but were visible in 73 % of 287 assayed dead eggs (*χ* ^2^ = 216, df = 1, *p* < 0.001; Fig. 4b), which mainly occurred in assayed dead eggs from the colder nil.7C-2d and nil.7C-5d treatments. However, visible larvae were largely absent from fumigated eggs, irrespective of whether they were assayed alive or assayed dead. There was no difference between the 25- and 45-g fumigation rates in the proportions of eggs that contained visible larvae (*χ* ^2^ = 0.01, df = 1, *p* = 0.94; Fig. 4b).

#### Egg turgidity

Figure 4c shows the Experiment 2 frequencies of eggs that exhibited differing levels of turgidity, versus time after fumigation, for living and dead eggs exposed to different concentrations of fumigant. Egg turgidity also exhibited a pattern similar to egg color. In the controls, egg turgidity was predominantly normal in assayed alive eggs, and predominantly intermediate in assayed dead eggs. However, fumigated eggs belonging to the assayed alive and assayed dead groups mainly had normal turgidity, irrespective of fumigation rate (*χ* ^2^ = 2.9, df = 1, *p* = 0.09; Fig. 4c). Gross changes in egg turgidity generally did not become evident until at least several days after eggs had died, and normal turgidity persisted in some inferred dead eggs even beyond 40 days (Fig. 4c).

### Survival analysis based on morphological criteria

Figure 2b shows Kaplan–Meier survival curves obtained when egg viability was estimated using morphological criteria. Comparison with results from biochemical assays (Fig. 2a) shows that estimates of delays between treatment and 100 % mortality obtained from morphological assessments were at least double those obtained from biochemical assays. An exception was the 25g.20C treatment, where both assessment methods gave similar survival curves (Fig. 2), but this only occurred because too few eggs were available from this treatment to continue monitoring their morphology after 100 % mortality had been confirmed with the biochemical assay.

## Discussion

Our results confirmed earlier indications that methyl bromide is slow acting. We measured delays to ≥95 % mortality of *M. domestica* eggs that ranged from 7.8 to 27.5 days. This range corresponded with post-fumigation survival of *A. ludens* larvae for up to 7 days (Hallman and Thomas 2011), *C. pomonella* embryos for up to 15.5 days (Yokoyama et al. 1988), and *H. lataniae* adults for up to 31 days (Witherell 1984). It also corresponded with the 9 days required for ATP to decline by 97 % in fumigated *A. cervinus* eggs (Forney et al. 1991).

Post-fumigation delays to *M. domestica* mortality were 1.5–2.5 times longer when eggs were stored at 10–11 °C rather than 20–21 °C; this is consistent with fumigation efficacy being enhanced by temperatures that promote insect metabolic activity (Bond 1984). However, our result that delay to mortality is influenced by storage temperature is new because it differs from previous observations that temperature influences mortality rate (Bond 1984).

*Musca domestica* would have received relatively high effective doses of methyl bromide in our experiments because we fumigated the eggs in open Petri dishes in the absence of any plant material. In normal phytosanitary fumigations, effective doses can be limited by a range of factors including sorption of methyl bromide by plant material (Bond 1984) and by imperfect penetration of methyl bromide to pests situated within plant tissue (Williamson et al. 1986). The high effective doses delivered by our experimental conditions suggest our estimates of delays to mortality could be conservatively short. Nevertheless, additional experiments involving fumigations of plant products infested by regulated pests are needed to clarify how closely our results from fumigating a model organism under artificial conditions apply to real-world situations.

The observed negative relationship between storage temperature and delay to mortality might break down at temperatures outside those tested in our study. Fumigated and control eggs stored at 7 °C for 5 days showed similar delays to mortality, probably because *M. domestica* eggs have low tolerance to cool temperatures (Leopold 2000), so the relationship we observed could reverse at even cooler storage temperatures. A similar effect occurred with a tenebrionid beetle because its median lethal methyl bromide dose increased as temperature declined to 10 °C, then decreased again between 10 and 0 °C, presumably due to the additional lethal effect of cold temperature (Shepard and Buzick 1939). The reversal might also happen at high storage temperatures; Armstrong and Whitehand (2005) found that fumigation efficacy against two tephritid flies increased from 15 to 25 °C, then declined at higher temperatures.

Our results and previous experiments (Forney et al. 1991; Hallman and Thomas 2011; Witherell 1984; Yokoyama et al. 1988) indicate that many different species and life stages die slowly after methyl bromide fumigation, and we suspect this also occurs with other treatments. We tested numerous arthropod species while validating a biochemical viability assay (Phillips et al. 2013) and obtained preliminary evidence of delays to mortality that exceeded 7 days after treatments that included several insecticide sprays and phosphine fumigation.

Hallman and Thomas (2011) showed that inspections of grapefruit can occur before all larvae of a fruit fly (*A. ludens*) have succumbed to methyl bromide fumigation. In New Zealand, rock melons imported from Australia are inspected 2 days after fumigation if they are air-freighted, and 5–7 days after fumigation if they are sea freighted; so our results also indicate that delays to mortality will sometimes exceed the intervals between methyl bromide fumigation and inspection.

Our study showed that there are two reasons why inspections could reveal “live” specimens in commodities that have received effective doses of methyl bromide: delays to mortality, and dead insects retaining the appearance of living specimens. The second of these phenomena was probably caused by methyl bromide’s antimicrobial properties (Schmittle 1955; Munnecke et al. 1959; Harry et al. 1972; Menge et al. 1978) inhibiting degradation of dead eggs. It is possible that our efforts to maintain the eggs in a sterile environment to optimize their viability exacerbated this effect, but it is unlikely to fully account for it. Petri dishes containing eggs were always placed in fumigation chambers without their lids, so eggs would have been fully exposed to airborne microbes while loading and unloading the chambers. Also, control eggs, which received similar microbe exposures to fumigated eggs, showed postmortem abnormalities in color and turgidity more often and more quickly. Thus, using turgidity and color to assess egg viability during phytosanitary inspections could frequently lead to the incorrect conclusion that methyl bromide fumigation has been ineffective.

The presence of visible larvae within *M. domestica* eggs responded differently to methyl bromide fumigation than other morphological characters. Larvae were visible inside control eggs more often than in fumigated eggs, probably because fumigation disrupted embryonic development (Yokoyama et al. 1988). However, larvae were also visible within dead control eggs more often than in live control eggs, probably because live eggs hatched so quickly that developing larvae were infrequently observed. Larvae were most often seen within control eggs that had died during storage at 7 °C. Therefore, the absence of visible larvae in dead and live fumigated eggs was partly confounded by their rarity in live control eggs.

The central issue for our morphological assessments was that morphological signs of death became evident long after mortality had occurred. However, we did not fully implement MPI’s morphological assessment protocol because we did not slide mount eggs to look for embryo movement using a compound microscope. Would looking for embryo movement have alleviated this issue? The answer is ‘no’, because MPI’s protocol states that an immobile embryo in an otherwise healthy looking egg should be classified as live. Moreover, Yokoyama et al. (1988) observed that some embryos continued development for up to 15.5 days after fumigation, which indicates that some treated embryos could remain capable of movement; this would only exacerbate concerns about treatment efficacy.

The biochemical viability assay (Phillips et al. 2013) had advantages over morphological assessments because it detected mortality sooner after treatment. However, even this method is inadequate for verifying the phytosanitary safety of commodities that are inspected before all pests have succumbed to a slow acting treatment.

New methods for verifying treatment efficacy would reduce economic losses and negative environmental impacts arising from unnecessary rejections of commodities that contain live, but lethally treated and dying pests. They could also assist in assuring the phytosanitary safety of irradiated commodities by providing alternatives to certification. Pests exhibit biochemical responses to treatments well before they die, and these could be exploited to develop new verification methods. For example, in our experiments, *M. domestica* ATP concentrations began to decline immediately after eggs were fumigated, but they slowly increased in live control eggs stored at 10 and 20 °C (Fig. 3). Commodities exhibit biochemical responses to treatments as well, and these also offer new possibilities for verifying efficacy, as was recently illustrated by a proof of concept test for verifying various foods have been heat treated (Iline et al. 2013).

Biochemical methods for verifying live pests have been lethally treated would have different benefits to biochemical methods for verifying commodities have been treated to standard. A verification test for a live pest would provide confidence both that treatments were effective and post-treatment reinfestation had not occurred, but phytosanitary safety would remain partly dependent on the efficacy of inspections for detecting pests, which could be low (Work et al. 2005). Verification tests for commodities would circumvent the need to inspect for live pests, thus mitigating risks associated with imperfect detection of pests, but might not provide assurance against post-treatment reinfestation. Despite these potential shortcomings, both approaches would help to reduce unnecessary rejections of commodities that contain live, but lethally treated and dying pests.
